# Evaluating the relationship between the 4-item migraine interictal burden scale and other patient-reported outcome measures: a *post hoc* analysis from a phase 3 migraine prevention study

**DOI:** 10.3389/fneur.2026.1808619

**Published:** 2026-06-05

**Authors:** Christian Lampl, Richard B. Lipton, Sarah Lipsius, Janet H. Ford, Grazia Dell’Agnello, Lars Viktrup, Dawn C. Buse

**Affiliations:** 1 Hospital Barmherzige Brüder Linz, Linz, Austria; 2 Albert Einstein College of Medicine, Bronx, NY, United States; 3 Montefiore Medical Center, Bronx, NY, United States; 4 Syneos Health, Morrisville, NC, United States; 5 Eli Lilly and Company, Indianapolis, IN, United States

**Keywords:** correlation, disability, interictal burden, migraine, patient-reported outcomes, quality-of-life

## Abstract

**Background:**

People with migraine report reduced health-related quality of life during ictal and interictal phases. However, most patient-reported outcome measures (PROMs) focus on impact during migraine episodes (ictal) or globally (both ictal and interictal), potentially neglecting interictal burden. This exploratory analysis evaluated correlations between the 4-item Migraine Interictal Burden Scale (MIBS-4) total score and other PROMs in people with migraine.

**Methods:**

A *post hoc* analysis was conducted using participant data from a galcanezumab phase 3, placebo-controlled, 3-month study, followed by a 3-month open-label extension in participants with prior treatment failures. Correlations were assessed between baseline MIBS-4 and disease characteristics, baseline MIBS-4 and demographics, and MIBS-4 and other PROMs/monthly migraine headache days at baseline and at Month (M) 6, using Spearman’s rank correlation coefficient (r_s_).

**Results:**

A total of 462 participants were included (mean age: 45.8 years); 85.9% were female, 58.2% had episodic migraine and 41.8% had chronic migraine. At baseline, highest correlations with MIBS-4 were observed for Migraine Specific Quality of Life Questionnaire (MSQ)-total score, MSQ-Emotional Function (MSQ-EF), and the Patient Health Questionnaire (PHQ-9) score, which measures depressive symptoms (all *p* < 0.001). Correlation analysis between MIBS-4 and MSQ-total score revealed moderate correlation at baseline (*r*_s_:–0.53) that transitioned to moderate-high at M6 (*r*_s_:–0.70). MIBS-4 was moderately correlated with PHQ-9 score at baseline (*r*_s_:0.55) and M6 (*r*_s_:0.55). The Migraine Disability Assessment score transitioned from moderate-low correlation at baseline (*r*_s_:0.41) to moderate at M6 (*r*_s_: 0.53) and Generalized Anxiety Disorder score had moderate-low correlation at both time points (*r*_s_:0.42–0.47). MIBS-4 had moderate-negligible correlation with monthly migraine headache days at baseline (*r*_s_:0.21) and low correlation at Month 6 (*r*_s_:0.32).

**Conclusion:**

Interictal burden, as assessed by MIBS-4, was moderately correlated with PROMs like MSQ, PHQ-9, and MIDAS, but moderate-negligible to low correlation was observed with monthly migraine headache days. These results indicate that interictal burden is a unique construct that is correlated with, but not fully captured by, other measures and should be considered when managing people with migraine.

**Clinical trial registration:**

ClinicalTrials.gov identifier NCT03559257.

## Background

Migraine is a highly prevalent neurological disorder and remains a major leading cause of disability burden globally (1–3). Most patient-reported outcome measures (PROMs) assessing the impact of migraine, focus on ictal burden (during the migraine attack) or on the overall impact of migraine (4–6). However, studies have shown that individuals with migraine also report reduced health-related quality of life (HRQoL) and poorer subjective well-being during the interictal phase (migraine-related impairment between migraine episodes) (1, 7, 8). It is estimated that 26–60% of people with migraine experience interictal burden (9, 10). Interictal burden may limit participation in activities, making plans, or fulfilling commitments due to concerns and anxiety about future migraine episodes, often resulting in avoidance behaviors (8, 10). Moreover, some symptoms such as cognitive impairment, photophobia, and allodynia may persist interictally, limiting overall quality of life (1, 2, 7, 8).

One measure specifically designed to determine the degree of interictal burden is the 4-item Migraine Interictal Burden Scale (MIBS-4) which assesses disruption at work and school, impairment in family and social life, difficulty making plans or commitments, and emotional/affective and cognitive distress (1, 11). Validation testing of the scale found a positive association between interictal burden and psychological symptoms (e.g., anxiety and depression), reduced workplace productivity, migraine-related stigma, and worsening scores on various measures of migraine disability (e.g., Migraine Disability Assessment Scale [MIDAS], Headache Impact Test [HIT-6]), migraine attack frequency, and Migraine-Specific Quality of Life Questionnaire [MSQ] (1, 11–13).

The CONQUER study was the first to use the MIBS-4 to assess the impact of a migraine preventive treatment on interictal burden (14–16). Results demonstrated that galcanezumab significantly reduced migraine headache days as well as interictal burden (14, 16). These reductions were significant in both episodic (EM) and chronic migraine (CM) subpopulations (16). Including multiple PROMs in the CONQUER study enabled a cross-sectional *post hoc* analysis to examine interictal burden by assessing correlations between the MIBS-4 and other PROMs. Although the MIBS-4 exhibits strong face validity and test–retest reliability, its ability to capture constructs distinct from those assessed by conventional migraine outcome measures remains to be established. Given the increasing time constraints faced by clinicians, patients, and researchers, it is essential to determine whether a separate assessment of interictal burden contributes meaningful additional information to the evaluation and management of migraine.

We hypothesized that MIBS-4 would have a modest positive correlation with many outcome measures. This result would support the validity of MIBS and also help to establish that it makes a unique contribution to the overall assessment of migraine burden. This exploratory *post hoc* analysis also aimed to determine whether the uniqueness of interictal burden measurement persisted over 6 months of active treatment.

## Methods

### Study design

Data for this post hoc analysis were drawn from a phase 3, multicenter, randomized, double-blind, placebo-controlled study in people with EM or CM who had experienced treatment failure with 2–4 standard-of-care migraine preventive medications due to insufficient efficacy and/or safety/tolerability reasons within the last 10 years (CONQUER, ClinicalTrials.gov identifier: NCT03559257). A detailed description of the study design has been published (14, 15). Briefly, the CONQUER study consisted of an initial screening period, a one-month prospective baseline period, a 3-month, double-blind treatment period, and a 3-month open-label extension. Participants had to have at least 4 migraine headache days per month and at least 1 headache-free day during the prospective baseline period to be eligible. Baseline migraine frequency status was categorized as EM or CM, with EM defined as <8 migraine headache days per month or <15 headache days per month during the baseline period, and with CM defined as ≥15 headache days per month, of which at least 8 were migraine headache days, during the baseline period. Eligible participants were randomized 1:1 to receive double-blind monthly subcutaneous injections of galcanezumab 120 mg (with a loading dose of 240 mg; administered as two 120 mg injections) or placebo. All participants received galcanezumab 120 mg/month during the open-label treatment period (14).

### Study assessments

Participants used a daily electronic diary (e-diary) to record headache-related information, including headache occurrence, severity, features, and if any acute headache medication was taken. Starting at randomization, participants also completed various self-report scales at the monthly visits, including the MIBS-4 (1) and the MSQ version 2.1 (17). Other PROMS collected at baseline, Month 3, and Month 6, included: the MIDAS (18), Patient Global Impression of Severity (PGI-S; for overall migraine disease severity) (19), 9-item Patient Health Questionnaire (PHQ-9; for depression) (20), 7-item Generalized Anxiety Disorder scale (GAD-7) (21), and the Work Productivity and Activity Impairment Questionnaire (WPAI) (22). Further details for each of these scales are provided in Supplementary Appendix I.

### Statistical analyses

Baseline data for demographics, baseline disease characteristics, and outcome measures were pooled across the randomized participants in both treatment arms (galcanezumab and placebo; total study population) and also reported for the EM and CM subgroups. The total study population included participants randomized to the galcanezumab or placebo groups for the 3-month double-blind phase. In the 3-month open-label extension, all participants received galcanezumab. Eligible participants received at least one dose of the study drug.

Correlational analyses were conducted between the baseline MIBS-4 total score and baseline characteristics and baseline scores of outcome measures in the total study population and EM and CM subgroups. Correlational analyses were also conducted between these measures using data collected at Month 6, once all participants had the opportunity to receive at least 3 months of open-label galcanezumab. Correlations between the MIBS-4 total score and baseline disease characteristics and PROMs were conducted using the non-parametric Spearman’s rank correlation coefficient (r_s_), as many variables were not normally distributed (e.g., MIDAS total score) and/or were interval ordinal, or dichotomous (e.g., presence or absence of acute headache medication overuse). The Spearman’s rank correlation coefficient measures the strength and direction of association between two ranked variables, with values ranging from −1 to +1, and assesses the degree of a monotonic relationship between two variables rather than assuming a linear relationship, which is less restrictive (23). Positive correlations indicate directional agreement, in which higher (worse) MIBS-4 scores are associated with higher (worse) scores on measures of migraine burden, except for the MSQ total score (for which higher scores are better). Similarly, a positive correlation would result if higher (worse) MIBS-4 scores were associated with higher values for patient characteristics or other variables we analyzed. Lower values would correspond similarly. Negative correlations indicate that higher (worse) MIBS-4 scores are associated with lower (better) scores, or values on measures of migraine burden and patient characteristics, respectively. Conversely, lower (better) MIBS-4 scores are associated with higher scores/values on migraine burden measures and patient characteristics.

Correlations between the MIBS-4 total score and disease characteristics, demographics and PROMs were evaluated at baseline and depicted in a forest plot, which also displayed 95% confidence intervals and level of statistical significance. Correlations between the MIBS-4 total score and other PROMs/monthly migraine headache days/symptom-free headache-free days were evaluated at baseline and Month 6 in the total study population and individuals with EM or CM. No formal interaction testing was performed to assess whether correlations differed significantly between EM and CM subgroups; accordingly, observed differences in correlation coefficients between subgroups should be interpreted as descriptive and hypothesis-generating rather than inferential. To describe the strength of correlations between MIBS-4 and other PROMs, the following categories of Spearman’s rank correlation coefficient were utilized: 0- ≤ 0.2 = “negligible,” > 0.2- ≤ 0.3 = “moderate-negligible,” > 0.3- ≤ 0.4 = “low,” > 0.4- ≤ 0.5 = “moderate-low,” > 0.5- ≤ 0.6 = “moderate,” > 0.6- ≤ 0.7 = “moderate-high,” > 0.7- ≤ 0.9 = “high.” These granular categories facilitate refined interpretation while remaining anchored to conventional thresholds. Heat maps were used to display correlation coefficients at baseline and Month 6 in a matrix where the correlation for each pair of variables was color-coded to indicate the strength of correlation using the above-defined categories.

Among those treated with galcanezumab during both study phases, the cumulative proportion of participants achieving pre-defined response criteria for MIBS-4 and other key measures of migraine burden was summarized each month (Month 1 through Month 6) and displayed using a step graph to illustrate change over time. Additional details are provided in Supplementary Appendix II.

Statistical significance was declared based on a two-sided, 0.05 significance level (i.e., *p* < 0.05). Given the exploratory nature of our analyses, correlations were not corrected for multiple testing. While this approach increases the risk for Type I errors, we prioritized identifying potential associations for further study. Analyses were conducted using SAS Enterprise Guide 7.2 (SAS Institute Inc., Cary NC).

## Results

### Demographics and baseline characteristics

A total of 462 participants were randomized and received at least 1 dose of study treatment. Baseline characteristics for the total study population and EM and CM subgroups are reported in Table 1. Majority of the participants were female (85.9%) and white (81.7%). The mean age was 45.8 years, and study participants had an average of 4.2 comorbidities. At baseline, participants had an average of 13.2 monthly migraine headache days (EM subgroup: 9.3; CM subgroup: 18.7). Mean MIBS-4 total score was 5.5, indicating severe interictal burden (EM subgroup: 5.0; CM subgroup: 6.2). Average baseline MIDAS scores indicated severe migraine-related disability across groups. The MSQ total score indicated significant impairment in quality of life, with greater impairment (lower mean score) in the CM group than in the EM group. Overall, baseline demographic and disease characteristics were well balanced across treatment groups for the total population and EM and CM subpopulations, as previously reported (15).

**Table 1:** Baseline demographics and disease characteristics.

	**Total population** **(*N* = 462)**	**Episodic migraine (*N* = 269)**	**Chronic migraine** **(*N* = 193)**
Age in years, mean (SD)	45.8 (11.8)	46.1 (11.5)	45.3 (12.4)
Female, *n* (%)	397 (85.9)	229 (85.1)	168 (87.1)
White, *n* (%)^a^	365 (81.7)	233 (88.6)	132 (71.7)
Comorbidities, mean (SD)	4.2 (3.7)	4.0 (3.8)	4.4 (3.6)
Monthly migraine headache days, mean (SD)	13.2 (5.9)	9.3 (2.8)	18.7 (4.7)
Monthly migraine headache hours, mean (SD)	83.3 (71.1)	53.1 (35.2)	125.4 (85.8)
Monthly days with acute headache medication use, mean (SD)	12.3 (6.0)	9.5 (3.7)	16.2 (6.4)
MIBS-4 total score, mean (SD)^b^	5.5 (3.5)	5.0 (3.4)	6.2 (3.5)
MSQ total score, mean (SD)^c^	52.0 (17.4)	54.8 (16.4)	48.1 (18.1)
MIDAS total score, mean (SD)^d^	50.9 (45.7)	39.3 (30.6)	67.2 (57.0)
PGI-S, mean (SD)^e^	4.6 (1.2)	4.4 (1.1)	4.9 (1.3)
PHQ-9, mean (SD)^f^	7.8 (5.3)	6.7 (4.7)	NA
GAD-7, mean (SD)^g^	5.0 (4.2)	4.6 (4.0)	5.5 (4.4)
WPAI^h^ Absenteeism (% time), mean (SD)	10.3 (19.2)	8.5 (16.0)	13.0 (22.9)
WPAI^h^ Presenteeism (% time), mean (SD)	42.7 (23.5)	39.0 (25.2)	48.3 (19.6)
Monthly symptom-free, headache-free days, mean (SD)	14.3 (6.2)	NA	NA

GAD-7, 7-item Generalized Anxiety Disorder scale; MIBS-4, 4-item Migraine Interictal Burden Scale; MIDAS, Migraine Disability Assessment Scale; MSQ, Migraine-Specific Quality of Life Questionnaire; PGI-S, Patient Global Impression of Severity; PHQ-9, 9-item Patient Health Questionnaire-9; SD, standard deviation; WPAI, Work Productivity and Activity Impairment Questionnaire.^a^ Race was reported by 447 (total population), 263 (episodic migraine), 184 (chronic migraine) individuals; Comorbidities were reported by 379 (total population), 203 (episodic migraine), 176 (chronic migraine) individuals; WPAI outcomes were reported for absenteeism by 326 (total population), 193 (episodic migraine), 133 (chronic migraine) individuals and for presenteeism by 320 (total population), 192 (episodic migraine), 128 (chronic migraine) individuals.^b^The MIBS-4 consists of four items reflecting interictal burden over the past 4 weeks. The total MIBS-4 score can range from 0 to 12, with higher scores indicating worse interictal burden (1).^c^The MSQ v2.1 consists of 14 questions reflecting the impact of migraine on health-related quality of life over the past 4 weeks, across three domains: role function-restrictive, role function-preventive, and emotional functioning. The total MSQ scores can range from 0 to 100 with higher scores indicating better health-related quality of life ((17)).^d^The MIDAS consists of five items reflecting disability over the past 3 months. The total MIDAS score can range from 0 to 90, with higher score indicating more disability (18).^e^The PGI-S measures the severity of illness and was collected every 3 months. The PGI-S includes a range of possible responses, from 1 (“normal, not at all ill”) to 7 (“extremely ill”) (19).^f^The PHQ-9 consists of nine items reflecting symptoms of depression plus an item assessing functional impact over the past 2 weeks. The total PHQ-9 score can range from 0 to 27, with higher scores representing more severe depression (20).^g^The GAD-7 consists of seven items reflecting symptoms of anxiety over the past 2 weeks. The total GAD-7 score can range from 0 to 21, with a total score of ≥10 indicating the possible presence of an anxiety disorder (21).^h^The WPAI consists of six items reflecting overall work impairment, absenteeism, presenteeism, and non-work activity impairment over the past 7 days attributable to a specific health problem. The WPAI scores are expressed as impairment percentages, with higher numbers indicating more significant impairment, i.e., worse outcomes (22).

### Correlations between MIBS-4 total score and disease characteristics and between MIBS-4 total score and demographics at baseline

**Figure 1:**
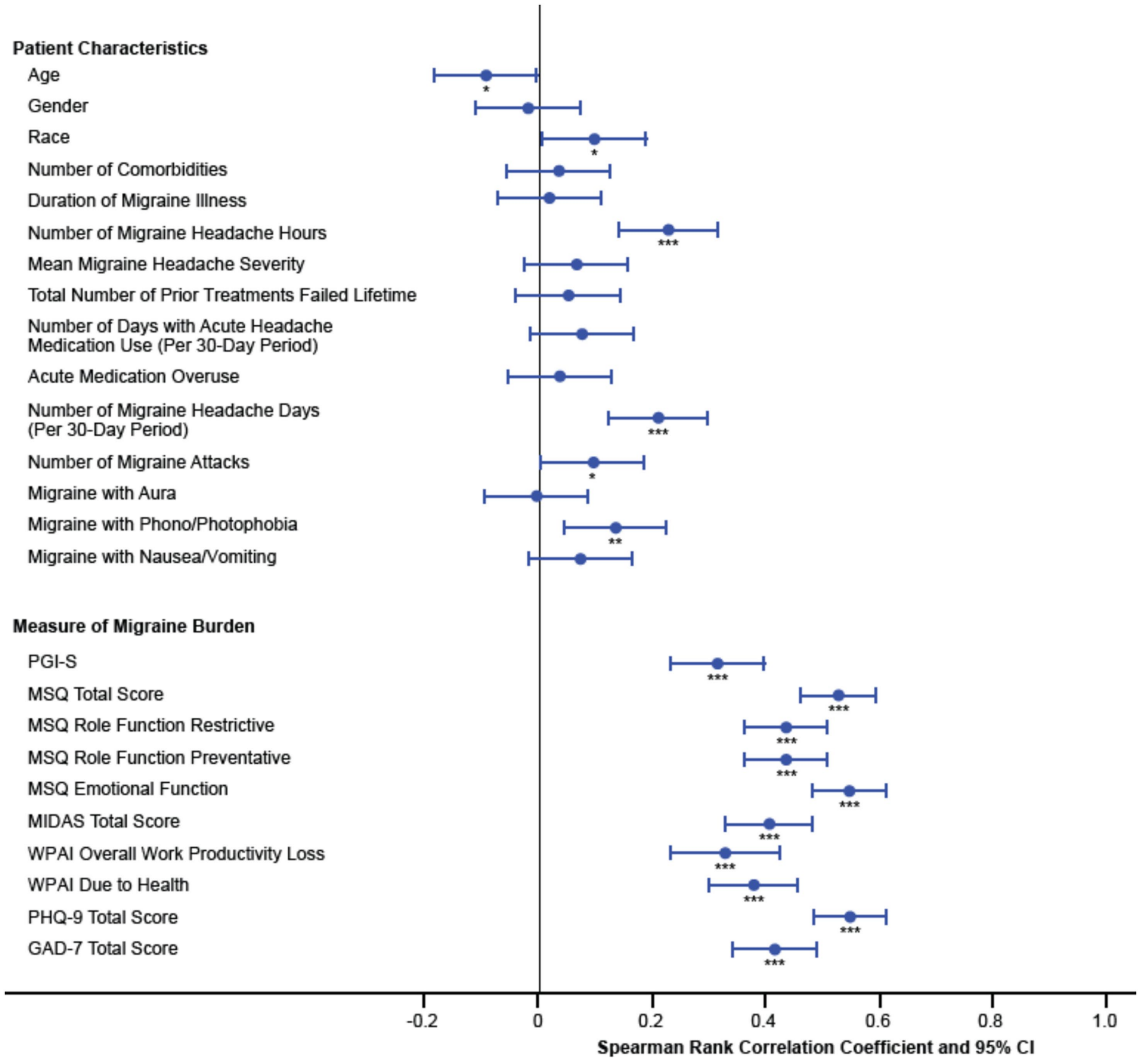
Forest plot of correlations between MIBS-4 total score and disease characteristics, demographics, and PROMs at baseline – total study population. CI, confidence interval; GAD-7, 7-item Generalized Anxiety Disorder scale; MIBS-4, 4-item Migraine Interictal Burden Scale; MIDAS, Migraine Disability Assessment; MSQ, Migraine-Specific Quality of Life Questionnaire; PGI-S, Patient Global Impression of Severity; PHQ-9, 9-item Patient Health Questionnaire-9; PROMs, patient-reported outcome measures; WPAI, Work Productivity and Activity Impairment Questionnaire.For this figure only, the MSQ scores are reverse-coded so that higher values indicate worse migraine-related quality of life. This aligns its directionality with the other measures of migraine burden for which higher scores reflect greater impairment. Positive correlations indicate directional agreement, where higher (worse) scores on the MIBS-4 correspond to higher (worse) scores on the measures of migraine burden, or higher values for patient characteristics. Lower values would correspond similarly. Negative correlations indicate that higher (worse) scores on the MIBS-4 are associated with lower (better) scores or lower values for measures of migraine burden and patient characteristics, respectively. And, conversely, lower (better) scores on the MIBS-4 are associated with higher scores on migraine burden measures and patient characteristics.Number of people with migraine included in the analysis: *N* = 462 for all except race (*N* = 447) and WPAI overall work productivity loss (*N* = 326).Non-numeric variables were recoded as follows: gender (1 = male, 0 = female); race (1 = white, 0 = non-white); medication overuse (yes = 1, no = 0); migraine with aura (yes = 1, no = 0); migraine with phono/photophobia (yes = 1, no = 0), migraine with nausea/vomiting (yes = 1, no = 0).Comorbid conditions (other than migraine) were recorded in medical history and adverse events data, and only those that began prior to, and ended after, first dose of study drug were counted as comorbidities. Those participants with zero comorbid conditions were included in the correlation analysis (*N* = 462).Duration of migraine illness (in years) was calculated relative to first dose of study drug. Migraine headache severity was collected daily in the electronic patient-reported outcomes diary and recorded as 1 = mild, 2 = moderate, 3 = severe. The mean migraine headache severity at baseline was calculated for each participant based on the daily observations.Spearman’s rank correlation coefficient categories were defined as: 0–≤0.2 = “negligible,” > 0.2–≤0.3 = “moderate-negligible,” > 0.3–≤0.4 = “low,” > 0.4–≤0.5 = “moderate-low,” > 0.5–≤0.6 = “moderate,” > 0.6–≤0.7 = “moderate-high,” > 0.7–≤0.9 = “high.”* *p* < 0.05, **p < 0.01, *** *p* < 0.001.

At baseline, moderate-negligible correlations (0.2 < *r*_s_ ≤ 0.3) with the MIBS-4 total score were observed for migraine headache duration, monthly frequency of migraine headache days, and migraine with phono/photophobia. MIBS-4 total score had negligible correlation (*r*_s_ ≤ 0.1) with parameters related to age, gender, race, number of comorbidities, prior duration of migraine, mean migraine headache severity, number of prior preventive medication categories failed, acute headache medication use/overuse, number of migraine attacks, migraine with aura, and migraine with nausea/vomiting (Figure 1).

### Correlations between MIBS-4 total score and other PROMs/monthly migraine headache days/symptom-free headache-free days throughout the study

When evaluating the correlation between the MIBS-4 total score and other PROMs at baseline, the highest correlations were observed for MSQ total score, MSQ-EF, and PHQ-9 total score (*r*_s_: 0.53–0.55) (Figure 1). Other PROMs showed lower, but statistically significant (*p* < 0.001) correlations with MIBS-4 total score, including MSQ-RFR, MSQ-RFP, MIDAS, and GAD-7 (*r*_s_: 0.41–0.44) (Figure 1).

**Figure 2:**
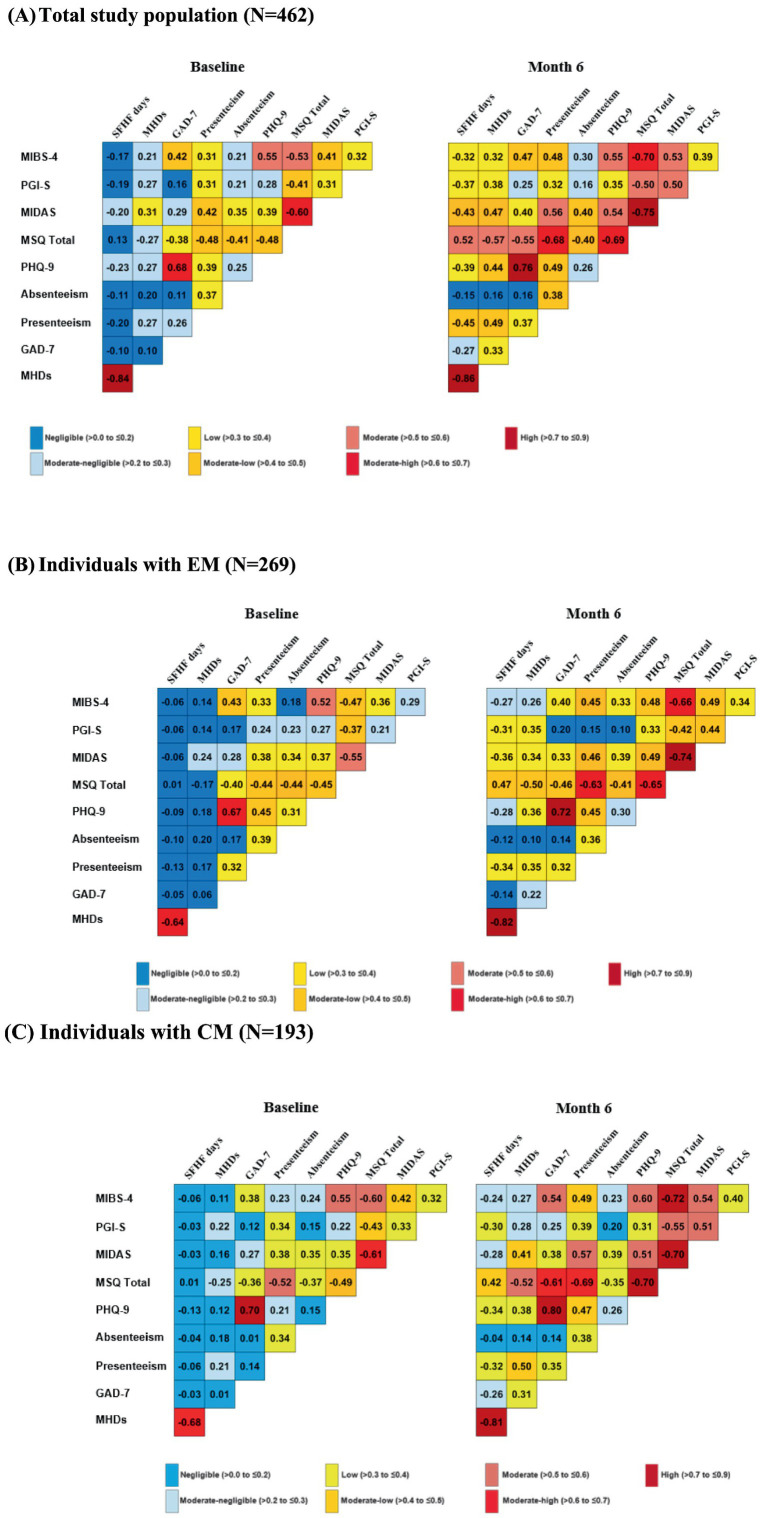
Heat maps showing Spearman’s rank correlation coefficients between PROMs and monthly migraine headache days at baseline and month 6. CM, chronic migraine; EM, episodic migraine; GAD-7, 7-item Generalized Anxiety Disorder scale; MHD, migraine headache days; MIBS-4, 4-item Migraine Interictal Burden Scale; MIDAS, Migraine Disability Assessment Scale; MSQ, Migraine-Specific Quality of Life Questionnaire; N, number of people with migraine included in the analysis; PGI-S, Patient Global Impression of Severity; PHQ-9, 9-item Patient Health Questionnaire; SFHF, symptom-free headache-free; WPAI, Work Productivity and Activity Impairment Questionnaire.Heat maps are presented in matrix form, where each cell represents the Spearman correlation coefficient between the row and column variables. Cell color corresponds to the magnitude of the correlation, as indicated in the figure’s legend. Positive correlations indicate directional agreement, where higher (worse) scores on the MIBS-4 are associated with higher (worse) scores on measures of migraine burden, or higher values of other variables (e.g., MHD’s), except for MSQ total score. Lower values would correspond similarly. The MSQ total score is coded in the opposite direction such that a higher score is better, therefore, a negative correlation between MIBS-4 and MSQ total score indicates that a higher (worse) MIBS-4 score is associated with a lower (worse) MSQ total score and, conversely, a lower (better) MIBS-4 score corresponds to a higher (better) MSQ total score. For the remaining variables, a negative correlation with MIBS-4 indicates an inverse relationship such that a higher (worse) MIBS-4 score is associated with a lower (better) value for the other variable and vice versa.Number of people with migraine included in the analysis at baseline (total study population): *N* = 462 for all except WPAI absenteeism (*N* = 326) and WPAI presenteeism (*N* = 320). Number of people with migraine included in the analysis at month 6: *N* = 431 for MIBS-4, MSQ total, MHD, and SFHF days, *N* = 428 for PGI-S, MIDAS, PHQ-9, and GAD-7, *N* = 279 for WPAI-absenteeism, *N* = 278 for WPAI-presenteeism.Number of people with EM included in the analysis at baseline: *N* = 269 for all except WPAI absenteeism (*N* = 193) and WPAI presenteeism (*N* = 192). Number of people with EM included in the analysis at month 6: *N* = 256 for MIBS-4, MSQ total, MHD, and SFHF days; *N* = 255 for PGI-S, MIDAS, PHQ-9, and GAD-7; and *N* = 171 for WPAI absenteeism and WPAI presenteeism.Number of people with CM included in the analysis at baseline: *N* = 193 for all except WPAI absenteeism (*N* = 133) and WPAI presenteeism (*N* = 128). Number of people with CM included in the analysis at month 6: *N* = 175 for MIBS-4, MSQ total, MHD, and SFHF days; *N* = 173 for PGI-S, MIDAS, PHQ-9, and GAD-7; *N* = 108 for WPAI absenteeism; and *N* = 107 for WPAI presenteeism.

The strength of correlations between MIBS-4 total score and other PROMs/monthly migraine headache days/symptom-free headache-free days at baseline and Month 6 are shown in the heatmap (Figure 2). At baseline, MIBS-4 showed moderate correlations with PHQ-9 and MSQ total score, and moderately low correlations with GAD-7 and MIDAS. By Month 6, the correlation of MIBS-4 total score with the MSQ total score and the MIDAS transitioned to moderate-high and continued to be moderately correlated with PHQ-9. In addition, MIBS-4 continued to have a moderately low correlation with GAD-7 scores, while presenteeism (from WPAI) transitioned from a low correlation at baseline to a moderate-low correlation at Month 6. Of note, MIBS-4 had moderate-negligible or low correlation with monthly migraine headache days at baseline and Month 6. In general, correlations in the EM and CM subgroups were similar to those observed in the total study population. Of note, the relationship between MIBS-4 and MSQ total score at baseline was weaker (r_s_: −0.47, moderate-low) in those with EM compared to the total study population (*r*_s_: −0.53, moderate; Figure 2).

Results for the cumulative proportion of individuals with migraine achieving response criteria for interictal burden and key measures of migraine burden at each month from Month 1 through Month 6 are provided in Supplementary Appendix II. The proportion of participants meeting the responder criteria increased over 6 months of treatment across all outcomes studied. Responder rates at 6 months were highest for MIBS (73.2%), followed by monthly migraine headache days, number of days of acute medication use per 30-day period and MSQ-RFR.

## Discussion

The present *post hoc* analysis leveraged the inclusion of multiple PROMs in the CONQUER study to characterize the interictal burden using the MIBS-4 scale and to evaluate its correlation with disease characteristics, demographics, and other established PROMs in people with migraine. Results from this analysis indicate very weak correlations between the MIBS-4 total score and disease characteristics, and between the MIBS-4 total score and baseline demographics. However, interictal burden, as assessed by MIBS-4, was moderately correlated with other PROMs capturing the impact of migraine episodes on HRQoL of people with migraine, especially MSQ. MIDAS seemed less correlated to MIBS-4 than MSQ. Interestingly, we only found a negligible to low correlation between interictal burden and monthly migraine headache days, suggesting it is not the number of migraine headache days themselves that is associated with the severity of the interictal burden.

While evaluating correlations between baseline MIBS-4 total score and baseline characteristics and demographics, MIBS-4 was not correlated with prior duration of migraine illness and number of previous treatment failures, and a very weak correlation was observed with migraine headache days. When assessing correlations between the MIBS-4 total score and PROMs at baseline, the highest correlations were observed with the MSQ total score, MSQ-EF domain, and PHQ-9 total score. Over 6 months, the correlation between interictal burden and PROMs appeared to change. At baseline, the MSQ total score showed a moderate correlation with MIBS-4, but this transitioned to a moderate-high correlation at Month 6. The MSQ was designed to measure both the ictal and interictal burden of migraine, so this correlation would be expected. Additionally, the similar recall period for the MIBS and MSQ scales, which is 4 weeks, might have positively influenced the correlation between the two PROMs. The MIDAS had a moderate-low correlation with MIBS-4 at baseline, which strengthened to a moderate correlation at Month 6. Moderate positive correlation of MIBS-4 with MIDAS observed in our study is in line with previously reported results (4). The difference in correlation of MSQ and MIDAS to MIBS-4 may be due to the fact that MIDAS is primarily a measure of ictal impairment whereas the MSQ may capture more of a global (ictal and interictal) burden. All other measures had negligible to moderate-low correlations with MIBS-4. In general, the degree of correlation between MIBS and other PROMs was similar across the total study population and subgroups, including those with EM and CM (Figure 2). The low correlations observed at baseline across multiple PROMs could be attributed to a heterogeneous population (with regard to disease burden). Improvements across all PROMs were observed in patients receiving galcanezumab and correlations strengthened over time, which may be consistent with reduced variability in migraine burden during the study period. At baseline, none of the participants had received treatment, whereas by 6 months, all had received treatment, potentially leading to a more homogeneous response. Additionally, a possible transition from CM to EM in a subset of participants and loss to follow-up among those with more heterogeneous clinical presentations may have further reduced variability, resulting in stronger correlations at follow-up. However, these observed changes in correlations during the treatment period cannot establish a treatment effect. Of note, the emotional functioning aspect of quality of life takes time to adjust, as observed in our study. This study also found that symptoms of anxiety, as assessed by GAD-7, were positively correlated with MIBS-4 total score. This is to be expected since anxiety as a trait measured in the GAD-7 and as a state or a reaction as measured in the MIBS-4 would be expected to be highly correlated.

Our study reported moderate-negligible to low correlation between interictal burden and monthly migraine headache days. These results may seem counterintuitive to observations from other studies that report a stronger correlation between MIBS-4 and monthly migraine headache days (9, 10, 12, 24). While interictal burden generally increases with the number of migraine headache days/month (9, 12, 25, 26), a recent study of 500 people with migraine (250 people each with EM and CM) found that although people with CM reported greater severity of interictal burden, based on MIBS-4 scores, than those with EM, headache frequency was not among the 37 variables that were found to contribute to predicting interictal burden using Machine Learning including Random Forest plot (27). This may be caused by the nonlinear relationship between headache frequency and interictal burden. Someone with very infrequent migraine (e.g., once a year) may not worry about the next attack, and someone with predictable daily migraine has little or essentially no interictal time, and therefore, little interictal burden. They know what to expect, even if it is dire, and they plan or do not make plans accordingly. However, these findings should be interpreted with caution and explored further given the multidimensional nature of migraine burden and the possibility that even modest correlations may reflect clinically relevant relationships.

Although the Spearman correlation coefficient describes the direction and strength of the association, squaring the Spearman correlation coefficient provides an estimate of the proportion of variance shared between the ranked variables. For example, the correlation between MIBS-4 and MSQ total score at baseline was −0.53, indicating that approximately 28% of the variance in their ranked scores is shared, while the remaining 72% is unexplained by this association. A shared variance of 28% indicates a moderate association; the variables share a meaningful but not overwhelming portion of their rank variability. This supports that MIBS-4 is a distinct yet related construct to quality of life, capturing unique aspects of interictal burden not fully captured by PROMs, focusing on the migraine attack. Clinicians may find MIBS-4 valuable in routine practice for identifying patients with a significant interictal burden. High rates of interictal burden may indicate a need for improved treatment optimization which can include optimizing acute therapy and starting or optimizing preventive therapy. High rates of interictal burden are also associated with greater stigma, anxiety, depression and worse quality of life. Theoretically reducing interictal burden may be associated with a range of positive outcomes. Routine screening for interictal burden using MIBS-4 could improve shared decision-making and patient satisfaction by targeting this underrecognized migraine dimension.

This study has limitations. The analysis was restricted to individuals who failed multiple preventive treatments and had ≥4 migraine headache days per 30-day period, limiting generalizability. As these analyses were cross-sectional, we cannot determine directionality. A major limitation of this analysis is the reliance on unadjusted Spearman correlations, which do not adjust for potential confounders (e.g., migraine severity, psychological comorbidities, treatment exposure) or effect modifiers that may influence the observed strength of association. Multivariable regression or partial correlation analyses would better isolate independent predictors of interictal burden. While this exploratory analysis demonstrated correlation between MIBS-4 score and other PROMs, it does not confirm construct independence as moderate correlations suggest meaningful shared variance and due to potential conceptual overlap. Future studies employing factor analysis or structural equation modeling could further delineate the unique interictal burden construct. In addition, this analysis includes limitations common to self-reported data, including recall and response biases. Varying recall periods across different PROMs may limit the interpretation of correlations. Conducting sensitivity analysis in future studies could help determine how the differences in recall periods may affect the correlations observed in this study. Furthermore, even though this is a comprehensive evaluation of essential PROMs, other variables that were not captured in this study may be associated with interictal burden. However, the inclusion of multiple PROMs, assessment at a baseline and subsequent 6-month follow-up period highlights the impact of interictal burden on a patient’s quality of life. Lastly, as the findings are not based on hypothesis testing, they should be interpreted with caution. Future studies will be necessary to validate these preliminary findings.

In conclusion, the correlation of the MIBS-4 total score and monthly migraine headache days transitioned from moderate-negligible to low over time, indicating that typical clinical trial endpoints, such as migraine headache days and monthly migraine days, do not fully capture the burden of migraine. This study shows that interictal burden is a distinct outcome for individuals with migraine and requires the utilization of a PRO instrument that is specifically designed to measure this outcome. Overall, there is value for researchers and clinicians to assess multiple outcomes, specifically recognizing that there is a disease burden between migraine episodes that needs to be addressed when evaluating and treating people with migraine.

## Data Availability

Lilly will provide access to anonymized individual participant data collected during the study. The data will be available to request on vivli.org after the study team has completed analyses and publications. Access will be provided after a proposal has been approved by an independent review committee identified for this purpose and after receipt of a signed data sharing agreement. After a proposal is approved, data and documents, including the study protocol, will need to be provided in a secure data sharing environment. For details on submitting a request, see the instructions provided at www.vivli.org.
