# Supplementary material for: Evaluating the relationship between the 4-item migraine interictal burden scale and other patient-reported outcome measures: a post hoc analysis from a phase 3 migraine prevention study
Source: Front Neurol. 2026 Jun 5;17:1808619. doi: 10.3389/fneur.2026.1808619 (PMC13281396; doi:10.3389/fneur.2026.1808619)
Supplement: Supplementary file 1 [file Supplementary_file_1.docx]

# Supplementary Appendix I

## Daily Headache Diary

Using an electronic diary, individuals reported daily headache-related information that included headache occurrence, intensity, features, and if and how long (number of days) any acute headache medication was taken. Information was also collected regarding migraine-associated symptoms, such as photophobia, phonophobia, nausea, and/or vomiting. An algorithm was used to identify a migraine headache day as a calendar day on which the criteria for migraine or probable migraine were met. Data obtained on acute headache medication taken were analyzed to determine whether medication overuse had occurred. The definition of acute headache medication overuse and other parameters related to migraine headache were based on criteria adapted from Section 8.2 of the ICHD-3 guidelines (1). Medication overuse at baseline was defined as present if a participant exceeded the following thresholds for total days of use of any of the following medications/drug classes per 30-day period of the prospective baseline period: any triptans, ergots or ergotamine derivatives, opioids, or barbiturates for ≥10 days; any non-steroidal anti-inflammatory drugs or aspirin, or acetaminophen/paracetamol for ≥15 days; and any use of 2 or more of the above drug classes on a given day for ≥10 days, either through individual medications or through fixed combination medications ≥2 of the 6 classes above. Daily entries were recorded for a 30-day baseline period, throughout the 3-month double-blind and 3-month open-label phase of the trial.

## The 4-item Migraine Interictal Burden Scale (MIBS-4)

The MIBS-4 is a patient-rated scale that evaluates the impact of headaches on individuals during the interictal period or times without headache. Questions include: item 1, “My headaches affect my work or school at times when I do not have a headache”; item 2, “I worry about planning social or leisure activities because I might have a headache”; item 3, “My headaches impact my life at times when I do not have a headache”; item 4, “At times when I do not have a headache, I feel helpless because of my headaches” (2). The recall period for MIBS-4 is 4 weeks, during which individuals provide responses specific to headache-free days. Each question has 6 possible responses: “don’t know/not applicable,” “never,” “rarely,” “some of the time,” “much of the time,” and “most or all of the time”. Responses to each of the four items were assigned scores as follows: 0=“don’t know/not applicable or never,” 1= “rarely”; 2=“some of the time”; 3=“much of the time or ‘most or all of the time”. Scores for each of the four items were then summed to obtain a total score that ranged from 0-12 (with higher scores indicating worse interictal burden). Categorical levels of interictal burden based upon the total score have been defined as: 0, “none”; 1-2, “mild”; 3-4, “moderate” and 5-12, “severe” (2). The MIBS-4 has been validated to show a moderate correlation with patient-reported outcomes of migraine-related disability, migraine-specific HRQoL impairments, workplace productivity, anxiety, and depression) (2) and had a 2-week test-retest reliability of 0.68 (3, 4). Validation of the severity categories from the MIBS-4 and relative to the MIDAS have been demonstrated (3). The MIBS-4 was collected at baseline and monthly through Month 6.

## Migraine Disability Assessment

The MIDAS is a patient-rated scale that quantifies headache-related disability over 3 months (4). This instrument consists of five items that reflect the number of days reported as missed or with reduced productivity at work/school or home and social events. Each question is answered as the number of days during the past 3 months of assessment, ranging from 0-90, with the total score being the summation of the 5 numeric responses. A higher value is indicative of more disability. The MIDAS was collected at baseline, Month 3, and Month 6.

## Patient Global Impression of Severity (PGI-S)

The PGI-S scale (5) is a patient-rated instrument that measures illness severity. For this study, the participant was instructed as follows: “Considering migraine as a chronic condition, how would you rate your level of illness?” The PGI-S includes a range of possible responses, from 1 (“normal, not at all ill”) to 7 (“extremely ill). The PGI-S was collected at baseline, Month 3, and Month 6.

## Migraine-Specific Quality of Life Questionnaire (MSQ) v2.1

The MSQ is a patient-rated scale of 14 questions (6). The questions measure the impact of migraine on HRQoL over the past 4 weeks across 3 domains: 1) Role Function-Restrictive (7 questions), examines the degree to which performance of daily activities is limited by migraine; 2) Role Function-Preventive (4 questions), examines the degree to which performance of daily activities is prevented by migraine; 3) Emotional Functioning (3 questions), examines feelings of frustration and helplessness due to migraine. Responses are given using a 6-point Likert-type scale, where 1=“none of the time,” 2=“a little bit of the time,” 3=“some of the time,” 4=“a good bit of the time,” 5=“most of the time,” and 6=“all of the time,”. Raw scores for each domain are computed as a sum of item responses, with the collective sum providing a total raw score that is then converted to a 0-100 scale, with higher scores reflecting better HRQoL. The MSQ was collected at baseline and monthly through Month 6.

## The 9-item Patient Health Questionnaire (PHQ-9)

The PHQ-9 is a patient-rated instrument that measures depressive symptoms over the past 2 weeks and is aligned with the Diagnostic and Statistical Manual of Mental Disorders- 5th edition (7) criteria for major depressive disorder (8). The first nine items include questions about anhedonia; depressed mood; trouble sleeping; feeling tired; change in appetite; guilt, self-blame, or worthlessness; trouble concentrating; feeling slowed down or restless; and thoughts of being better off dead or hurting oneself, followed by a 10^th^ item which assesses the impact of symptoms on functioning. Each of the nine symptom items is rated on a four-point scale (0=“never,” 1=“several days,” 2=“more than half the time,” 3=“nearly every day”) based on symptoms over the past 2 weeks. The total PHQ-9 score can range from 0-27, with higher scores representing more severe depression. The possible presence of a major depressive disorder is indicated if five or more of the nine depressive symptom criteria are rated as occurring at least “more than half the time”, with at least one of these items being anhedonia or depressed mood, and if functioning is rated as “somewhat difficult,” “very difficult,” or “extremely difficult.” (8) The PHQ-9 was collected at baseline, Month 3, and Month 6.

## Generalized Anxiety Disorder 7-item Scale (GAD-7)

The GAD-7 is a patient-completed, seven-item questionnaire that assesses anxiety symptoms (feelings of nervousness, uncontrollable worrying, excessive worrying, trouble relaxing, restlessness, irritability, and fearfulness) over the past 2 weeks. Each of the seven items is rated on a four‑point scale (0=“not at all,” 1=“several days,” 2=“more than half the days,” 3=“nearly every day”) (9). The total GAD-7 scores range from 0-21, with higher total GAD-7 scores representing higher severity of anxiety. The possible presence of anxiety disorder is indicated if the total score is ≥10 (9). The GAD-7 was collected at baseline, Month 3, and Month 6.

## Work Productivity and Activity Impairment Questionnaire (WPAI)

The WPAI is a patient-rated, six-item questionnaire that assesses overall work impairment, absenteeism, presenteeism, and non-work activity impairment over the past 7 days attributable to a specific health problem (10), in this case, migraine. Of the four possible domains, absenteeism (missing work) and presenteeism (impaired productivity at work) were analyzed. Scores are expressed as impairment percentages, with higher numbers indicating more significant impairment, that is, worse outcomes. The WPAI was collected at baseline, Month 3, and Month 6.

# Supplementary Appendix II: Proportion of participants achieving response criteria for interictal burden and key measures of migraine burden in the group receiving galcanezumab

## Approach

The cumulative proportion of individuals with migraine achieving response criteria for MIBS-4 and the key measures of migraine burden were depicted from Month 1 through Month 6. People with migraine who received galcanezumab and entered the open-label period were included in this analysis. Response criteria for each of the measures were selected based on the possible meaningful change thresholds available in the published literature and were defined as follows:

- ≥50% reduction (EM) or ≥30% reduction (CM) in the number of days with acute headache medication use (monthly) (11);
- ≥50% reduction (EM) or ≥30% reduction (CM) in monthly migraine headache days (monthly) (11);
- ≥25.71-point increase in MSQ-Role Function-Restrictive (MSQ-RFR; monthly) (12);
- ≥20.0-point increase in MSQ-Role Function-Preventive (MSQ-RFP; monthly) (12);
- ≥26.67-point increase in MSQ-Emotional Function (MSQ-EF; monthly) (12); and
- ≥2-point decrease in MIBS-4 total score (monthly) (2).

For each of the key measure of migraine burden, a step graph of the cumulative percentage of participants meeting the response criteria at months 1 through 6 was presented. No attempt was made to identify the proportion of participants reaching responder status simultaneously for all 6 measures.

## Results

The cumulative proportion of individuals with migraine achieving response criteria for interictal burden and key measures of migraine burden at each month from Month 1 through Month 6 is depicted in **Figure S1**. At Month 1, except MSQ-EF, 46.0%–50.4% of participants treated with galcanezumab achieved response criteria for all variables, that is, MIBS-4, monthly migraine headache days, number of days with acute medication use per 30-day period, MSQ-RFR, and MSQ-RFP. During subsequent months, the proportion of people with migraine achieving respective response thresholds increased for all measures. At Month 6, response criteria for the 6 measures of migraine burden were achieved by approximately 60% to 80% of people. Specifically looking at the MIBS-4, the percentage of people with migraine who met response thresholds was 46.4% at Month 1 and 73.2% at Month 6. The proportion of people with migraine achieving respective response thresholds was most similar for 5 of the 6 measures. The only outlier was the MSQ-EF domain, with fewer participants meeting the response criteria at baseline (40%) and Month 6 (approximately 60%). Although the response rate increased during subsequent months, it still lagged behind other PROMs.

## Figure S1. Cumulative percent of responders by month for MIBS-4 total score and other key PROMs among patients who received galcanezumab during the double-blind treatment period and entered the open-label period

**Abbreviations:** MIBS-4, 4-item Migraine Interictal Burden Scale; MSQ-EF, Migraine-Specific Quality of Life Questionnaire-Emotional Function; MSQ-RFP, Migraine-Specific Quality of Life Questionnaire-Role Function-Preventive; MSQ-RFR, Migraine-Specific Quality of Life Questionnaire-Role Function-Restrictive; PROM, Patient-Reported Outcome measures.


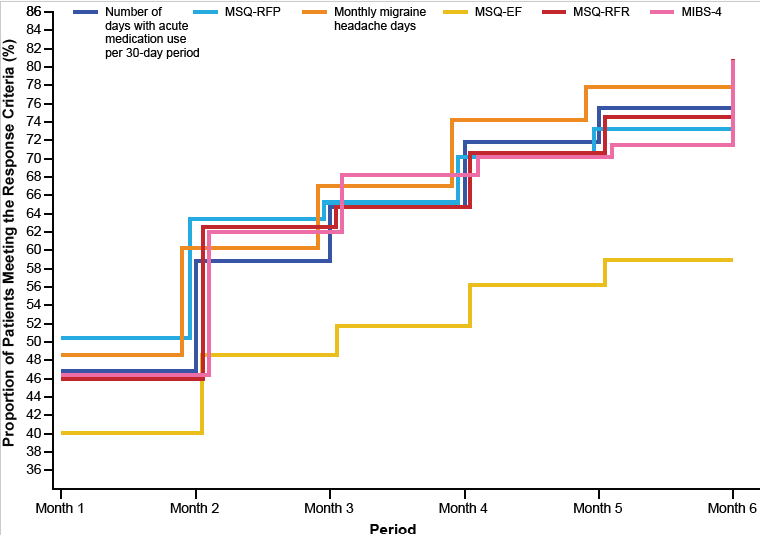


# References

1. International Headache Society (IHS). Headache Classification Committee of the International Headache Society (IHS) The International Classification of Headache Disorders, 3rd edition. *Cephalalgia* (2018) 38:1–211. doi: 10.1177/0333102417738202
2. Buse DC, Rupnow MFT and Lipton RB. Assessing and managing all aspects of migraine: Migraine attacks, migraine-related functional impairment, common comorbidities, and quality of life. Mayo Clin Proc. (2009) 84: 422–35. doi: 10.1016/S0025-6196(11)60561-2
3. Buse DC, Bigal M and Rupnow MF. The Migraine Interictal Burden Scale (MIBS): results of a population-based validation study [abstract F64]. Headache. (2007) 47:741–812. https://doi.org/10.1111/j.1526-4610.2007.00843.x
4. Stewart WF, Lipton RB, Kolodner K, Liberman J, and Sawyer J. Reliability of the migraine disability assessment score in a population-based sample of headache sufferers. Cephalalgia. (1999) 19:107–14. doi: 10.1046/j.1468-2982.1999.019002107.x
5. Guy W. ECDEU assessment manual for psychopharmacology. Rockville, MD: National Institute of Mental Health, Psychopharmacology Research Branch.1976; pp 217-222. Available at: https://archive.org/details/ecdeuassessmentm1933guyw
6. Bagley CL, Rendas-Baum R, Maglinte GA, Yang M, Varon SF, Lee J, et al. Validating Migraine-Specific Quality of Life Questionnaire v2.1 in episodic and chronic migraine. Headache. (2012) 52:409–21. doi: 10.1111/j.1526-4610.2011.01997.x
7. American Psychiatric Association, DSM-5 Task Force. (2013). Diagnostic and statistical manual of mental disorders: DSM-5™ (5th ed.). American Psychiatric Publishing, Inc. [https://doi.org/10.1176/appi.books.9780890425596](https://psycnet.apa.org/doi/10.1176/appi.books.9780890425596)
8. Kroenke K, Spitzer RL and Williams JB. The PHQ-9: validity of a brief depression severity measure. J Gen Intern Med. (2001) 16:606–13. doi: 10.1046/j.1525-1497.2001.016009606.x
9. Spitzer RL, Kroenke K, Williams JB, and Löwe B. A brief measure for assessing generalized anxiety disorder: the GAD-7. Arch Intern Med. (2006) 166:1092–7. doi: 10.1001/archinte.166.10.1092
10. Reilly MC, Zbrozek AS and Dukes EM. The validity and reproducibility of a work productivity and activity impairment instrument. Pharmacoeconomics. (1993) 4:353–65. doi: 10.2165/00019053-199304050-00006
11. Silberstein S, Tfelt-Hansen P, Dodick DW, Limmroth V, Lipton RB, Wang SJ, et al. Guidelines for controlled trials of prophylactic treatment of chronic migraine in adults. *Cephalalgia.* (2008) 28:484–95. doi: 10.1111/j.1468-2982.2008.01555.x
12. Speck RM, Yu R, Ford JH, Ayer DW, Bhandari R, and Wyrwich. Psychometric validation and meaningful within-patient change of the Migraine-Specific Quality of Life questionnaire version 2.1 electronic patient-reported outcome in patients with episodic and chronic migraine. *Headache.* (2021) 61:511–26. doi: 10.1111/head.14031
